# Pleomorphic circulating megakaryocytes in a case of CALR positive primary myelofibrosis in leukemic transformation

**DOI:** 10.1002/jha2.272

**Published:** 2021-07-29

**Authors:** Habib Moshref Razavi

**Affiliations:** ^1^ Division of Hematopathology Royal Columbian Hospital New Westminster British Columbia Canada; ^2^ Division of Hematopathology Department of Pathology and Laboratory Medicine University of British Columbia Vancouver British Columbia Canada

A case of 59‐year‐old female is described. Three years ago, being symptomatic with an enlarged left upper quadrant abdominal mass, she presented to a walk in clinic but eventually opted for alternative acupuncture treatment without imaging or a full blood count (FBC) at that time. With worsening bloating, early satiety and dyspepsia, she returned to her doctor where an ultrasound confirmed the presence of hepatosplenomegaly (with liver span of 27.5 cm and spleen size of 26 cm). An FBC showed an increase in white count with left shifted neutrophilia, basophilia and the presence of frank blasts (52.7, 17.9, 4.7 and 3.4 × 10^9^/L, respectively). Suspicious for a myeloproliferative neoplasm such as chronic myelogenous leukaemia, a bone marrow biopsy was organized and showed leucoerythroblastosis, bone marrow fibrosis (MF3) and osteosclerosis. Computed tomography imaging confirmed massive splenomegaly (30 cm). With a negative breakpoint cluster region protein‐abelson murine leukaemia viral oncogene homolog 1 (BCR‐ABL1) on peripheral blood fluorescence in situ hybridization, a presumptive diagnosis of primary myelofibrosis awaited the results of a janus kinase 2 (JAK2) status which was negative. In addition to a normal female karyotype by cytogenetics, an expanded myeloid mutation screen returned a TEIR I variant of strong clinical significance (additional sex combs like 1; *ASXL1* [variable allele frequency; VAF: 23.4%]). Tier III variants with mutations in calreticulin, *CALR* gene and src homology 2‐B adapter protein 3, *SH2B3* were also present (VAF 27% and 48.7% respectively). She was subsequently diagnosed with a *JAK2* negative primary myelofibrosis (dynamic international prognostic scoring system score‐4 and mutation enhanced international prognostic score [MIPSS70] score‐6). Her illness was confounded by cryptogenic cirrhosis status post‐banding of esophageal varices. She was treated with Ruxolitinib 10 mg p.o. daily until recently – where she has now become non‐compliant. A subsequent rise in the white count with an increase in blasts prompted a repeat biopsy leading to suspicion of an evolving leukaemic transformation (16% blood and 5% marrow blasts). Interestingly at this juncture, her peripheral blood film showed pleomorphic circulating megakaryocytes with mature morphology (Figure 1A‐E ×50 magnification), bare naked nuclei (Figure 1E‐G ×10 magnification) and immature megakaryoblasts (Figure 1H‐I ×10 magnification). Megakaryocytes are large polypoidal cells found within the bone marrow comprising 0.01% of all nucleated cells. They are known to physiologically migrate to the pulmonary circulation where locally platelets are released to the capillaries. For example, thick proplatelets but also megakaryocytes are known to be filtered and ensnared in microvascular bifurcations where turbulent flow and rich signaling milieu allows for generation of a platelet bioreactor. However, in normal subjects, demonstration of circulating megakaryocytes is exceedingly rare. Harbinger of extra medullary hematopoiesis, the finding is usually indicative of a serious disorder of the bone marrow. Specifically their presence is often accompanied with leucoerythroblastosis, and circulating blasts in such conditions as myelodysplasia, granulocytic leukemia, and myeloproliferative neoplasms such as myelofibrosis. Their recognition requires careful assessment of the entire blood film including the feathered edge. Owing to her liver cirrhosis and not being a candidate for a stem cell transplant, this patient has been deemed a candidate for palliative azacitidine therapy and is followed in the community.

## CONFLICT OF INTEREST

The author declares that there is no conflict of interest that could be perceived as prejudicing the impartiality of the research reported.

**FIGURE 1:**
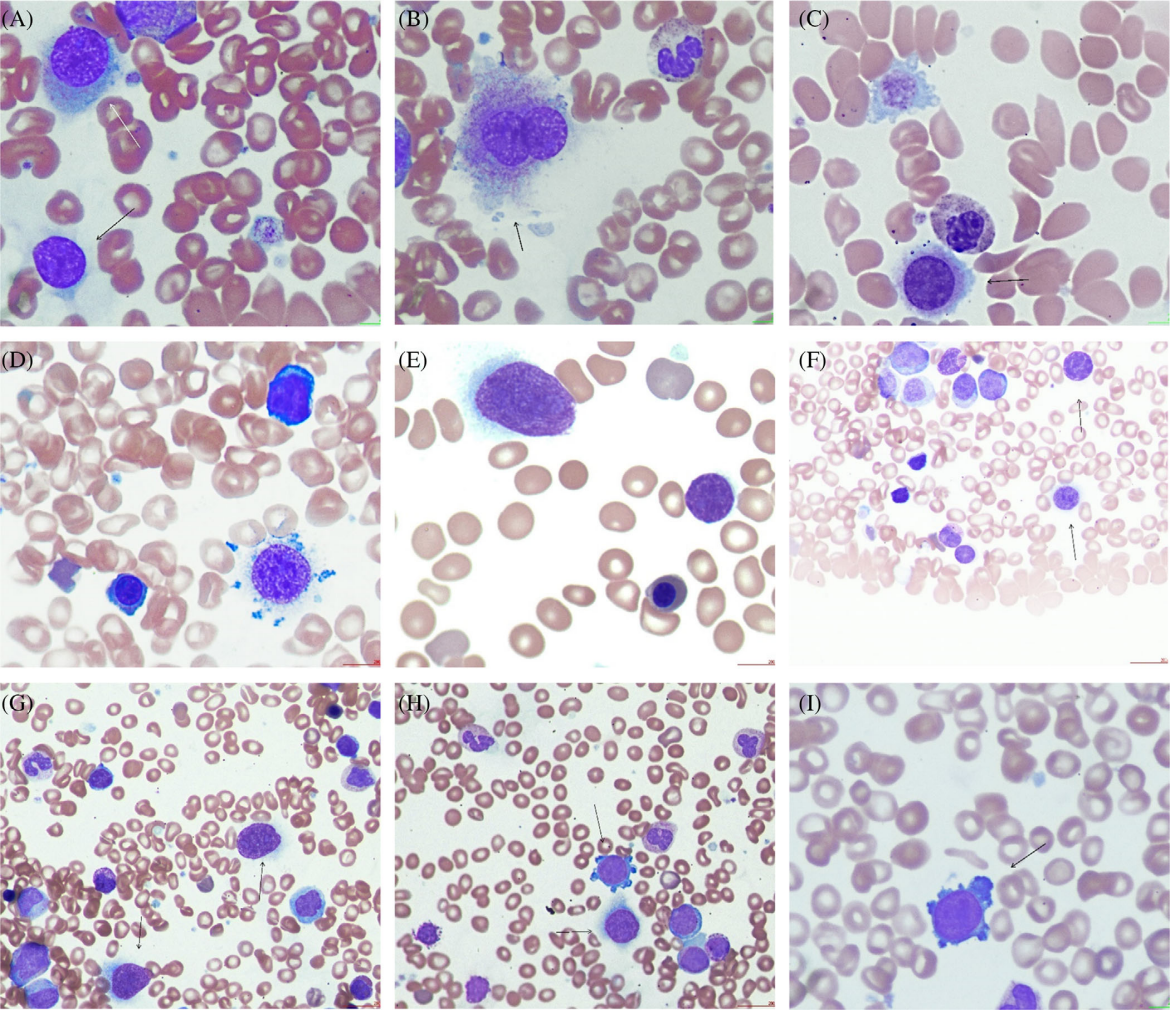
Pleomorphic circulating megakaryocytes in a case of CALR positive myelofibrosis in transformation is presented. (A‐C) Relatively mature circulating micromegakaryocytes. (D‐F) Barenaked nuclei in addition to above. (G and H) Occasional megakaryoblasts

